# The COVID-19 Prevalence among Children: Hypotheses for Low Infection Rate and Few Severe Forms among This Age Group in Sub-Saharan Africa

**DOI:** 10.1155/2021/4258414

**Published:** 2021-10-12

**Authors:** Sylvain Raoul Simeni Njonnou, Nadia Christelle Noumedem Anangmo, Fernando Kemta Lekpa, Diomede Noukeu Njinkui, Dominique Enyama, Christian Ngongang Ouankou, Eric Vounsia Balti, Esther Astrid Mbono Samba Eloumba, Jean Roger Moulion Tapouh, Simeon Pierre Choukem

**Affiliations:** ^1^Department of Internal Medicine and Specialties, Faculty of Medicine and Pharmaceutical Sciences, University of Dschang, Dschang, Cameroon; ^2^The University of Dschang Taskforce for the Elimination of COVID-19 (UNITED#COVID-19), Dschang, Cameroon; ^3^Department of Microbiology, Haematology and Immunology, Faculty of Medicine and Pharmaceutical Sciences, University of Dschang, Dschang, Cameroon; ^4^Douala General Hospital, Douala, Cameroon; ^5^Department of Pediatrics, Faculty of Medicine and Pharmaceutical Sciences, University of Dschang, Dschang, Cameroon; ^6^Douala Gynaeco Obstetric and Pediatric Hospital, Douala, Cameroon; ^7^Yaounde University Teaching Hospital, Yaounde, Cameroon; ^8^Diabetes Research Center and Department of Internal Medicine, Universiteit Ziekenhuis Brussel, Vrije Universiteit Brussel, Brussels, Belgium; ^9^Endocrine and Diabetes Unit and National Obesity Centre, Department of Internal Medicine, Yaounde Central Hospital, Yaounde, Cameroon; ^10^Department of Biochemistry, Faculty of Medicine and Biomedical Sciences, University of Yaounde I, Yaounde, Cameroon; ^11^Department of Radiology and Medical Imaging, Faculty of Medicine and Pharmaceutical Sciences, University of Dschang, Dschang, Cameroon; ^12^Health and Human Development (2HD) Research Network, Douala, Cameroon

## Abstract

Despite some cases of severe or critical manifestations of the coronavirus disease 2019 (COVID-19) described among children, the prevalence of this infection in the pediatric population is quite low worldwide, particularly in sub-Saharan Africa. Current data suggest indeed that, independent of the population considered overall, severe and critical cases of COVID-19 are rare among children. This observation prompted us to discuss the possible hypotheses which could explain the low prevalence of COVID-19 among children; amongst others, we discuss (1) immunomodulation by the Bacillus Calmette–Guerin vaccine or by some parasitic infections such as malaria, schistosomiasis, and helminthiasis and (2) cross immunization with other coronaviruses commonly found in the sub-Saharan African setting.

## 1. Introduction

The current pandemic of the coronavirus disease 2019 (COVID-19), caused by the Severe Acute Respiratory Syndrome Coronavirus-2 (SARS-CoV-2), originating from Wuhan in China has led so far to almost 240 million cases and more than 4,700,000 deaths worldwide [1]. So far, children have shown a low rate of infection as well as the low rate of severe or critical forms all over the world [2]. Although this age group is less tested than the others, and some severe or critical forms have been described in the literature, we are struck by the low number of infections or symptomatic forms among them [3, 4]. Thereby, less than 40% of children with a documented infection were symptomatic in Qatar where symptoms varied with the age [5]. This is even more marked in sub-Saharan Africa (SSA) [6], where, given the high rate of infectious and genetic comorbidities such as sickle cell disease, of this population, one would expect a greater prevalence as well as a greater number of deaths [7]. Of course, case fatality rates are low among children, probably due to fewer ACE2 receptors and other factors, but the rate seems to be lower in SSA [8, 9]. Immunomodulation is the modification of the immune response or the function of the immune system by substances that induce non-specific-antigen enhancement of the body's native or acquired defense mechanisms (immunomodulatory factors). We hypothesize that, in sub-Saharan African children, there may be some immunomodulatory factors that could lead to increased resistance of the immune system to COVID-19 and cross immunization thereby, explaining the low prevalence of COVID-19 among children.

## 2. The Impact of Intestinal Parasitic Infection on Immunity

The intestinal parasites, particularly helminths, are well-known immunomodulatory factors. Their regulatory role of the immune system has been documented in several inflammatory diseases such as severe asthma or multiple sclerosis [10]. These parasites are more frequent in low- and middle-income countries (LMICs) where inflammatory diseases are less prevalent than in developed countries. Moreover, they are more frequent in children than in adults. These helminths, which are multicellular parasites, are characterized by widespread downmodulation of both the innate and adaptive arms of host immunity. Thereby, the inflammatory pathways that are responsible for autoimmune or inflammatory diseases may be blocked by the presence of intestinal parasites, inducing an immunotolerogenic state [11].

Ssebambulidde et al. found an inverse correlation between the incidence of COVID-19 and soil-transmitted helminths [10]. These authors compared quantitative data on COVID-19-confirmed cases and deaths, extracted from the WHO Situation Report-104, as of May 04, 2020, to data collected from the 2018 schistosomiasis status data report for schistosomiasis cases and the 2018 soil-transmitted helminths report for soil-transmitted helminths cases. The European region had the highest proportion (45.34% of global COVID-19 cases and 59.79% of COVID-19 deaths). Europe had no schistosomiasis cases reported in 2018. Europe also had the lowest proportion (0.55%) of soil-transmitted helminth infections globally in 2018. Similarly, the Americas accounted for 41.34% of the global COVID-19 cases and 32.86% of COVID-19 deaths, with 0.41% of malaria cases, 0.71% of schistosomiasis cases, and 5.38% of the soil-transmitted helminths cases globally in 2018. Africa, otherwise, had the smallest proportion (0.88%) of COVID-19 cases and COVID-19 deaths (0.45%) globally, with the highest proportion (89.23%) of schistosomiasis cases, and the second-highest proportion (25.42%) of soil-transmitted helminths cases in 2018. The authors found that the median number of COVID-19 cases in the WHO African region was lower than that of the Eastern Mediterranean (153 vs. 2,344 cases, *p* < 0.001) and Europe (153 vs. 2,127 cases, *p* < 0.001) region. Furthermore, they found a negative correlation between COVID-19 and soil-transmitted helminth infections (*r* = −0.25, *p* < 0.001) and between COVID-19 and schistosomiasis (*r* = −0.38, *p* < 0.001). The authors also predicted the maximum number of COVID-19 cases for countries where schistosomiasis and helminthiasis are endemic, to less than 600 cases [10]. These predictions are, to date, far below the reality, suggesting the action of multiple factors and not only that of intestinal parasites.

## 3. The Impact of Intestinal Parasitic Infection on Immunity

Malaria is the commonest infection in children in SSA [12]. Children below 5 years account for the majority of death due to malaria in SSA. The disease prevalence in children in SSA is high as their immune system is immature [13]. If viral infections are known to induce immunomodulation in patients infected by *Plasmodium*, new data on immunomodulatory activity of *Plasmodium* on some viruses are available. For instance, patients with HIV infection or hepatitis B infection tended to present lower levels of parasitemia and decreased proinflammatory cytokine levels and were more likely to be asymptomatic in response to *Plasmodium* infection [14]. Paradoxically, *Plasmodium* parasitemia was associated with increased survival in Ebola virus-infected patients [15, 16], by promoting high interferon (IFN) response, which is impaired in patients with severe or critical COVID-19 forms [17].

Ssebambulidde et al. also compared quantitative data on COVID-19-confirmed cases and deaths, extracted from the WHO Situation Report-104 to data collected from the 2019 world malaria report for malaria-estimated cases and reported deaths in 2018. The authors found a negative correlation between COVID-19 and malaria (*r* = −0.17, *p*=0.02) [10]. Furthermore, because of the overlapping distribution of malaria and intestinal parasites, coinfection with both is common in SSA [18]. This coinfection could, therefore, reinforce the immunomodulation activity [14]. This hypothesis could explain why countries in SSA have fewer cases and deaths due to COVID-19 than others from northern Africa and South Africa, where malaria infection is almost eradicated. One concern that we should, however, raise is that, in some instances, similar to COVID-19, malaria infection could induce a cytokine storm.

To the best of our knowledge, we are not aware of any resistance of the *Plasmodium*-infected cell to COVID-19 either *in vivo* or *in vitro*. But, the enhanced IFN response is a pathway that needs to be explored by further studies.

## 4. Immunomodulation by Bacillus Calmette–Guerin Vaccination

Among the other immunomodulatory factors, we should cite vaccines, particularly the Bacillus Calmette–Guerin (BCG) vaccine. It is an attenuated *Mycobacterium bovis*, widely used in expanded immunization programs, particularly in SSA, as it confers cross protection for *Mycobacterium tuberculosis*. That vaccine does not only protect against tuberculosis. Epidemiological data showed a protective effect on respiratory tract infections (particularly viral lower tract infections) and improved survival in early childhood [19]. It has also been used to treat urothelial cancer. If BCG protection is established for children, it is inconsistent in adults. Furthermore, this protection is limited in populations with immunodeficiency syndromes. As the prevalence of tuberculosis decreased, some countries, including France, Germany, and Spain, stopped the mass vaccination of children and only perform it for high-risk subjects. Other countries, such as the United States of America, Italy, or Belgium, never established national universal BCG vaccination. This vaccine is widely used in SSA and administrated during the first few days after birth, to children through expanded immunization programs.

Escobar et al. collected data on COVID-19 mortality by country, to assess the effect of the BCG vaccination policy on COVID-19 mortality and assess linkages between the use of the BCG vaccine and the number of COVID-19 deaths. The authors found a consistent link between BCG vaccination and COVID-19 mortality under different scenarios [20]. Therefore, countries with a strong national BCG vaccination policy had lower COVID-19 deaths and those with the current BCG vaccination policy had lower COVID-19 deaths than those without or with an interrupted policy [20]. Interestingly, the BCG vaccine is associated with the enhancement of the IFN response [21]. However, mortality reduction in countries with BCG national universal policy is not sufficient to establish the causality of protection by BCG vaccine on severe or critical forms of COVID-19. These hypotheses deserve to be confirmed by prospective studies.

## 5. Cross Immunization with Other Viruses, Particularly Coronaviruses

Children are more prone to infections, especially viral infections. This proceeds to the education of their immune system. They can be infected by various types of viruses, especially those with respiratory tropism. Viral coinfections are frequent in children in SSA and can lead to death [22, 23]. As coronaviruses are present in many animals such as bats, civets, or camels, some strains of coronaviruses had been circulating in SSA prior to the COVID-19 pandemic [24]. Therefore, an infection of children by these strains is certain. Given this hypothesis, the infected children could develop a cross immunity against SARS-CoV-2. Then, children could develop immunity against this group of coronaviruses that could be crossed with that of COVID-19. Mateus et al. identified more than 140 human T-cell epitopes derived from across the genome of SARS-CoV-2. They provided evidence that numerous CD4+ T cells that react to SARS-CoV-2 epitopes are cross reacting to circulating common human coronaviruses [24].

This hypothesis is supported by data from a recent study. Tso et al. collected and tested pre-COVID-19 pandemic plasmatic samples from the USA and SSA by immunofluorescence assay against the spike and nucleocapsid proteins of all known human coronaviruses (HCoVs). They found a significantly higher prevalence of SARS-CoV-2 serological cross reactivity in samples from SSA compared to the USA. SARS-CoV-2 nucleocapsid proteins and spike proteins from other HCoVs were the most commonly recognized proteins in the cross reactive samples. This prepandemic serological cross recognition of HCoVs could contribute to explaining the low rate of SARS-CoV-2 infection and disease in SSA [25].

## 6. Impact of the Self-Prescription of Antimalarial Drugs

In SSA, parental self-prescription of antimalarial drugs to children is frequent. Mawili-Mboumba et al. found in a large cohort study in Gabon that, up to 21.4% of parents gave antimalarial drugs to their febrile children, even though 80% of these children were not infected by *Plasmodium* sp. [26]. Other authors found similar results in the other African countries [27, 28]. Even before the COVID-19 pandemic, antimalarial drugs were already known to have effects on respiratory viruses; nowadays, artemisinin-derived antimalarial drugs have been proposed as treatment options for COVID-19 [29, 30]. Subsequently, we may hypothesize that antimalarial self-medication may also affect pathogenesis and the clinical presentation of COVID-19 in children.

## 7. Childhood Obesity Association with COVID-19

Obesity has been identified as a risk factor for COVID-19 and COVID-19 death among the adult population [31]. Although to the best of our knowledge, there is no association between childhood obesity and COVID-19, it seems important to remember that childhood obesity is a powerful predictor of obesity in adulthood. Furthermore, it increases the risk of developing numerous health problems later in life, including diabetes and heart disease [32]. Therefore, there might be an association between childhood obesity and COVID-19.

Though SSA has been facing a dramatic increase in childhood obesity since 1990, the total number of overweight and obese children is still globally lower there than that in western countries [33]. We could, therefore, suggest that this lower prevalence of obesity in SSA could be one of the explanations for the fewer number of COVID-19 cases among children.

## 8. COVID-19 Undertesting Rate in SSA

One of the main challenges since the beginning of the pandemic, in SSA, was the case identification and ascertainment of this disease [34]. COVID-19 testing rates in SSA are the lowest worldwide. This is probably due to the cost of public health strategies for COVID-19 epidemic control [35]. In the setting of a limited health system, this financial burden is a major obstacle to the massive testing of the population. Furthermore, as children are less symptomatic than adults and there are less severe forms in this age group, the systematic use of screening tests is not respected.

## 9. Miscellaneous

Two other hypotheses, not directly linked to children, can be raised to explain the low prevalence of COVID-19 in this age group: firstly, the low infection rate among adults in SSA could be responsible for the low transmission rate among children; secondly, the reasons linked to SARS-CoV-2 itself (low viral load in infected patients or climatic conditions, which can reduce the virus transmission), which could explain this low prevalence of COVID-19 in SSA.

## 10. Conclusions

The prevalence of COVID-19 among children is low worldwide and much lower in SSA. Many hypotheses could sustain this observation. Among these, the action of immunomodulatory factors such as intestinal parasites, malaria infections, and BCG vaccine and cross immunization is the most seductive hypothesis and drives our attention. Some of these hypotheses could represent therapeutic pathways and need to be further explored in clinical trials.

## Data Availability

The data supporting this review are from previously reported studies and datasets, which have been cited. The processed data are available from Sylvain Raoul Simeni Njonnou upon reasonable request.
